# Reading motivation, self-regulated reading strategies and English vocabulary knowledge: Which most predicted students’ English reading comprehension?

**DOI:** 10.3389/fpsyg.2022.1041870

**Published:** 2022-12-15

**Authors:** Hong Li, Zhengdong Gan

**Affiliations:** ^1^School of Foreign Studies, Guangzhou University, Guangzhou, China; ^2^Faculty of Education, University of Macau, Taipa, Macao SAR, China

**Keywords:** reading motivation, self-regulated reading strategies, English vocabulary knowledge, English reading comprehension, self-regulated learning strategies

## Abstract

This study explored how reading motivation, self-regulated reading strategies and English vocabulary knowledge influenced students’ English reading comprehension simultaneously in one model. A total of 543 students from five universities in Southern China completed a reading motivation questionnaire, a reading strategy questionnaire, two vocabulary knowledge tests, and a reading comprehension test. Multiple regression analysis results showed that reading efficacy and enjoyment, and vocabulary knowledge (i.e., both vocabulary breadth and depth) significantly predicted reading comprehension. When students were grouped into high, average, and low achievers on the reading test, monitoring strategies and vocabulary depth were found to significantly predict reading comprehension for the high achievers.

## Introduction

Reading, as one of basic skills of a language, plays an indispensable role in second language acquisition. Naturally, L2 reading has attracted many researchers’ attention and an increasingly large body of research has explored aspects of L2 reading. These studies have attempted to unveil the secrets of how to become proficient L2 readers, with some examining the role of reading motivation (e.g., Shang, 2010; Kim, 2011; Lin et al., 2012; Dhanapala and Hirakawa, 2016; Hwang, 2019), some focusing on the role of reading strategies (e.g., Phakiti, 2003; Zhang and Zhang, 2013; Zhang et al., 2014; Guo, 2018), and others investigating the role of L2 vocabulary in developing reading comprehension (e.g., Qian, 1999, 2002; Kang et al., 2012; Rydland et al., 2012; Li and Kirby, 2015). While these studies have offered insight into our understanding of the different factors involved in reading comprehension, how reading motivation, self-regulated reading strategies, vocabulary knowledge may differentially contribute to L2 reading comprehension remains unclear.

Drawing upon the theoretical framework of self-regulated learning (SRL), the current study examined the joint influence of reading motivation, reading strategy use, and vocabulary knowledge on reading comprehension, which has not been researched in the prior research. The SRL model (Pintrich, 2000; Zimmerman and Schunk, 2011) considers learning as a constructive and active process, in which the learners set goals, and then regulate and manipulate their cognition, motivation, behaviors by considering social and contextual conditions. Relying on the SRL model outlined by Pintrich (2000) and Zimmerman and Schunk (2011), this study aims to address the following two research questions:

1. What may characterize Chinese university EFL students’ English reading motivation, reading strategies, and English vocabulary knowledge?
2. How do reading motivation, reading strategies and vocabulary knowledge contribute to students’ reading comprehension?

## Literature review

### Reading motivation

Reading motivation is defined as a certain kind of feeling which makes readers to be close to or refrain from a reading context (Readence et al., 1989). Guthrie and Wigfield (2000) viewed reading motivation as people’s own purposes, values and thoughts in relation to the themes, procedures, and outputs of reading. Previous studies have proposed different dimensions of reading motivation, and researchers generally agree that reading motivation is multidimensional. In the L1 reading context, many studies have proposed different models of reading motivation. For instance, Wigfield and Guthrie (1997) classified L1 reading motivation into “self-efficacy beliefs” (including reading efficacy and reading challenge), “intrinsic-extrinsic motivation and purpose for learning” (including curiosity, involvement, reading importance, reading avoidance, competition, recognition, and grades), and “social aspects of motivation” (including compliance and reading for social reasons; p. 420). Wang and Guthrie (2004) later established an intrinsic-extrinsic motivation model to explain reading motivation in the L1 context. Specifically, intrinsic motivation involves challenge, curiosity, and involvement, whereas extrinsic motivation involves grades, compliance, competition, social reasons, and recognition. Compared with L1 reading motivation, studies on the dimensions of L2 reading motivation are scant. Most of these studies have been conducted in Asia (e.g., Mori, 2002; Takase, 2007; Kim, 2011; Lin et al., 2012; Wang and Gan, 2021). While these researchers have identified different dimensions of L2 reading motivation in relation to their research contexts, they tend to agree that intrinsic motivation, extrinsic motivation and reading efficacy are the three most important types of reading motivation. With regard to mainland Chinese students’ English reading motivation, Wang and Gan (2021) have developed a reading motivation questionnaire in an English as a foreign language context (RMQ-EFL) which includes five dimensions, namely, reading efficacy, reading enjoyment, reading recognition, reading involvement, and reading compliance. The five dimensions of RMQ-EFL have their theoretical basis in the reading motivational constructs proposed by Wigfield and Guthrie (1997), Wang and Guthrie (2004), and Mori (2002).

In the English as a second language learning context, little attention has been given to the role of reading motivation in L2 reading comprehension. Previous studies generally have revealed a positive relationship between intrinsic reading motivation and reading comprehension (e.g., Kim, 2011; Lin et al., 2012; Dhanapala and Hirakawa, 2016). However, studies also show that intrinsic reading motivation does not correlate with L2 reading comprehension (e.g., Olmez, 2015; Park, 2015). Besides, mixed results have been found concerning the predicting role of extrinsic reading motivation in reading comprehension. Dhanapala and Hirakawa (2016) explored how reading motivation constructs are correlated with English reading text comprehension by investigating 406 Sri Lankan university students. Through structural equation modeling (SEM) analysis, Dhanapala and Hirakawa (2016) found that extrinsic motivation negatively linked with English reading comprehension. In another study with Korean EFL university students, Kim (2011) found learning goal-oriented motivation positively associated with English reading comprehension. Lin et al. (2012) study, instrumentalism, which represents extrinsic reading motivation, was found to be positively associated with EFL reading comprehension of the Hong Kong primary students. Additionally, the positive relationship between reading efficacy and reading comprehension has also been documented elsewhere (e.g., Shang, 2010; Irene, 2013; Boakye, 2015; Hwang, 2019), indicating a significant relationship of self-perceived reading ability with reading development. In sum, previous research generally confirm the positive effects of intrinsic reading motivation and reading efficacy on L2 reading comprehension. However, the predictive role of extrinsic motivation in L2 reading comprehension remains controversial. The role of L2 reading motivation in L2 reading comprehension, especially the role of extrinsic reading motivation in L2 reading comprehension, is yet to be further investigated.

Moreover, few studies have explored reading proficiency differences in the relationship between reading motivation and reading comprehension. Logan et al. (2011) investigated the differences between good and poor readers in terms of the role of intrinsic reading motivation in predicting reading comprehension and found intrinsic reading motivation only significantly predicts poor readers’ reading comprehension. McGeown et al. (2012) checked for differences between good and poor readers in the relationship between reading motivation (extrinsic and intrinsic motivation) and their reading comprehension. McGeown et al. (2012) found intrinsic reading motivation was not significantly associated with reading comprehension for both good and poor readers. McGeown et al. (2012) also showed extrinsic reading motivation significantly linked with good readers’ reading comprehension. However, the significant association between extrinsic reading motivation and reading comprehension was not observed among poor readers.

### Self-regulated reading strategies

#### Self-regulated learning

Self-regulated learning (SRL) refers to “learning that results from students’ self-generated thoughts and behaviors that are systematically oriented toward the attainment of their learning goals” (Schunk, 2001, p. 25). According to Pintrich and De Groot (1990), in the classroom context, self-regulated learning includes three important components: learners’ metacognitive strategies for planning, monitoring and modifying their cognition; the actual cognitive strategies learners adopt to learn, memorize, and understand the material; and learners’ management and control of their efforts on classroom academic tasks. In different models of SRL, self-regulated learners are usually depicted as being capable of manipulating cognitive, motivational, emotional, and behavioral areas of learning (Zimmerman and Schunk, 2011). For instance, Winne and Hadwin (1998) SRL model, which highlights the role of metacognition, emphasizes that self-regulated learners manage their learning activities through monitoring and mainly adopting metacognitive strategies (Winne, 1995, 1996; Winne and Hadwin, 1998). Meanwhile, Winne and Hadwin (1998) SRL model points to the goal driven nature of SRL and the possible impacts of self-regulated activities on motivation. In the academic literature, self-regulated learning is generally considered as an essential component for students’ reading development (Paris and Paris, 2001; Finkbeiner et al., 2012; Lau, 2012). Proficient readers are usually described as highly motivated self-regulated readers who can adopt a wide range of reading strategies in an effective way (Hilden and Pressley, 2007). In the current study, we look at how metacognitive and cognitive reading strategies contribute to students’ EFL reading comprehension.

#### Reading strategies

Afflerbach et al. (2008) described reading strategies as “deliberate, goal-directed attempts to control and modify the reader’s efforts to decode text, understand words, and constructs meanings of text” (p. 368). Studies generally confirm the positive role of reading strategies in L2 reading comprehension (e.g., Phakiti, 2003; Zhang and Zhang, 2013; Zhang et al., 2014; Guo, 2018). For instance, Phakiti (2003) investigated the association between test-takers’ reading strategy use and reading test achievement, and found that metacognitive and cognitive strategies were significantly and positively correlated with English reading test achievement, and that good readers used more metacognitive strategies compared with average readers, who used more metacognitive strategies compared with poor readers. Zhang and Zhang (2013) identified lexico-grammar and text comprehension as two factors that account for Chinese university students’ English reading achievement, and the former is significantly influenced by monitoring reading strategies. Zhang et al. (2014) found that reading test takers’ strategy use significantly affected their lexical and grammar knowledge and indirectly affected text comprehension through lexical and grammar knowledge. By investigating 268 sophomore students in a Southern Chinese university, Guo (2018) found that metacognitive strategy use, L1 reading proficiency, and L2 proficiency positively and directly predicted L2 reading comprehension and that metacognitive strategy use affected L2 reading comprehension through L2 proficiency and L1 reading proficiency. These studies generally show a positive role of reading strategies in reading comprehension among Chinese university students. Nevertheless, these studies mainly involved reading strategy factors and reading comprehension factors and neglected motivational variables and other forms of linguistic knowledge variables.

Meanwhile, some studies also showed no association between reading strategies and L2 reading comprehension. Alsamadani (2008) found that three types of reading strategies, namely, planning, attending, and evaluating, did not predict Saudi EFL students’ English reading comprehension. In Lindholm and Tengberg’s (2019) longitudinal study on Swedish L2 secondary students, no correlation was found between the reading comprehension and reading strategy use of female students. Finally, a small proportion of studies showed that reading strategies negatively predicted L2 reading comprehension. For instance, Jaekel (2020) found that language learning strategy use had a negative effect on an English language proficiency test.

Reading proficiency differences in relation to reading strategy use have also been explored in a number of studies (e.g., Rao et al., 2007; Zhang and Wu, 2009; Zhang, 2010), with researchers reaching the general consensus that good readers employ more reading strategies than do poor ones (e.g., Kozminsky and Kozmingsky, 2001). An example is Zhang and Wu (2009) who found that high reading proficiency students used significantly more global and problem-solving reading strategies compared with average or low reading proficiency students. Zhang (2010) found that strong and weak L2 readers had different amounts of metacognitive knowledge. By analyzing think-aloud data, Rao et al. (2007) found that good readers frequently adopted deep-level reading strategies (e.g., inferencing, reconstruction), whereas poor readers often used surface-level reading strategies (e.g., rereading, paraphrasing).

### Role of vocabulary knowledge in reading comprehension

Vocabulary knowledge can be divided into two aspects: breadth and depth. Breadth of vocabulary knowledge refers to knowing the fundamental uses of words, understanding written forms of words, and figuring out words’ meanings at the surface level (Nation, 2001). Depth of vocabulary knowledge refers to identifying the collocation, synonymy, and polysemy among English words (Read, 1995). Since the 1980s, many studies on L1 reading reveal that the breadth and depth of vocabulary play indispensable roles in reading comprehension (e.g., Anderson and Freebody, 1981; Beck et al., 1982). However, previous studies on L2 reading have mainly emphasized the role of vocabulary breadth in reading and have only begun to examine vocabulary depth in the late 1990s (Qian, 2002).

Regarding the role of vocabulary knowledge in L2 reading comprehension, different findings were generated in previous studies. First, vocabulary breadth and depth were found to have an equally important role in predicting L2 reading comprehension (e.g., Qian, 1999; Qian, 2002; Rydland et al., 2012). By recruiting 80 Chinese and Korean EFL students from two Canadian universities, Qian (1999) found that in addition to the predicting role of vocabulary breadth in English reading comprehension, vocabulary depth also predicted English reading comprehension uniquely. In Qian (2002) subsequent study, he recruited 217 L2 readers from different L1 backgrounds and found that depth plays a role equally important as that of breadth in predicting English reading comprehension. Nevertheless, Qian’s two studies both focused on L2 learners studying in Canada, thereby limiting the generalizability of his findings to the Chinese EFL context. Rydland et al. (2012) investigated 61 bilingual children in Norway and found that the breadth and depth of L2 vocabulary can explain the Woodcock Passage Comprehension (L2 reading comprehension) with a majority of variance. However, Rydland et al. (2012)study only recruited a small sample size, and many unexplained variances were observed in the global warming test, which functions as another type of L2 reading comprehension. Second, studies also showed that vocabulary breadth plays a more important role than vocabulary depth in predicting L2 reading comprehension. For instance, Li and Kirby (2015) explored the impact of vocabulary knowledge on English reading from the perspectives of breadth and depth by investigating 246 Grade 8 Chinese EFL students. They found that breadth and depth were moderately linked with each other and could affect word reading performance, but breadth showed a greater effect on word reading compared with depth. Finally, researchers also reported that depth plays a greater role than breadth in predicting L2 reading comprehension. For instance, Kang et al. (2012) argued that depth, rather than breadth, played a more important role in English reading comprehension. However, given that Kang et al.’s study focused on Korean students from an all-girls high school and involved a small sample size, further empirical work needs to be conducted to validate their findings.

Few studies have also investigated reading proficiency differences in the relationship between vocabulary knowledge and reading comprehension. Masrai (2019) investigated how differences in proficiency level affect the relationship between vocabulary knowledge and reading comprehension among adult EFL students in Saudi Arabia. Masrai (2019) found that only frequently used vocabulary words predicted weak readers’ English reading comprehension, whereas frequently used and less frequently used vocabulary words predicted strong readers’ English reading comprehension.

## Materials and methods

### Participants

A total of 543 undergraduate students from five universities in Southern China were recruited for this study. Among the five universities, one was a national key university, two were provincial key universities (i.e., universities that are under jurisdiction of the Ministry of Education), and two were provincial non-key universities (i.e., universities that are under jurisdiction of the local provincial governments). Table 1 displays the demographics of the sample. The students majored in different non-English majors, including computer science, mathematics, artificial intelligence, education, business management, international trade, accounting, and so on. These non-English major students were in their first or second year at the time of the study, and were aged from 16 to 22 years (*M* = 18.86, *SD* = 0.90). Among these students, 240 (44.2%) were male, and 303 (55.8%) were female. All participants had had at least 6 years of English language studies, and successfully passed the College Entrance Examination in China. These students’ scores in the English subject (maximum score = 150) of the College Entrance Examination ranged from 40 to 146, and their average score was 108. All first-year and second-year university students need to take College English as a compulsory course. They also need to pass the CET-4 in order to graduate and seek jobs.

**Table 1:** Demographics of the participants (*N* = 543).

	Total
Gender	
Female	303
Male	240
Year of study	
Year 1	276
Year 2	267
Majors	
Materials science	28
Energy and power	12
Mining and safety	11
Aeronautics and astronautics	10
Computer science	65
Business management	53
International trade	64
Artificial intelligence	12
Economic and trade	38
Sociology	39
Accounting	74
Education	87
Mathematics	50

### Instruments

#### English reading motivation questionnaire

We used Wang and Gan (2021) RMQ-EFL to investigate students’ English reading motivation. The questionnaire included 22 items (seven items for reading efficacy, six items for reading enjoyment, three items for recognition, three items for involvement, and three items for compliance). Each item was scored on a 4-point Likert scale ranging from 1 (very different from me) to 4 (a lot like me).

Wang and Gan (2021) questionnaire was used in this study for three reasons. First, this questionnaire had its theoretical origin in Wigfield and Guthrie’s (1997) and Wang and Guthrie’s (2004) reading motivational constructs. Second, the reliability and validity of this questionnaire were tested through EFA and CFA using robust psychometric measures. The CFA results for RMQ-EFL showed good model fit (*x*^2^/df = 1.49 < 3, CFI = 0.93, RMSEA = 0.06, SRMR = 0.06). The Cronbach’s alpha coefficients for five reading motivational sub-scales of RMQ-EFL are 0.90 (reading efficacy), 0.89 (reading enjoyment), 0.82 (recognition), 0.82 (involvement), and 0.74 (compliance). Third, the questionnaire was specifically designed for Chinese EFL readers and was validated through an empirical investigation in the Chinese EFL context.

#### Self-regulated reading strategy questionnaire

The Metacognitive and Cognitive Strategy Questionnaire (Zhang et al., 2014) was used to measure Chinese EFL learners’ self-regulated reading strategy use. The questionnaire included 38 items: 6 for planning, 8 for evaluating, 10 for monitoring, 3 for initial reading, 4 for identifying important information, 4 for integrating, and 3 for inference making. All items in this questionnaire were rated on a 6-point Likert scale, ranging from 0 (never) to 5 (always).

The Metacognitive and Cognitive Strategy Questionnaire was used in this study because of its theoretical lineage related to metacognition (e.g., Paris and Winograd, 1990; Wenden, 1998) and previous validation of this questionnaire with Chinese university EFL students (e.g., Zhang et al., 2014; Zhang, 2016; Zhang, 2018). The CFA results for the strategy questionnaire revealed acceptable model fit (*x*^2^/df = 1.70 < 3, CFI = 0.91, RMSEA = 0.03, SRMR = 0.05). The Cronbach’s alpha coefficients for the seven subscales of the strategy questionnaire were 0.62 (planning), 0.84 (evaluating), 0.79 (monitoring), 0.49 (initial reading), 0.56 (identifying important information), 0.70 (integrating), 0.67 (inference making). The overall reliability estimate of this questionnaire was 0.89.

#### English reading comprehension test

The English reading comprehension test in this study was a published version of the CET-4 reading subtest (June 2016 version). It comprised 30 items, including three sections: 10 vocabulary comprehension items, 10 long-form reading items, and 10 close reading items. The test had a total score of 35 points, of which 5 were for vocabulary comprehension (0.5 points for each correct answer), 10 were for long-form reading (1 point for each correct answer), and 20 were for close reading (2 points for each correct answer). This English reading comprehension test was adopted in this study for mainly two reasons. The CET-4 test has been specifically designed for Chinese university EFL students, and this test has gone through a rigorous validation process (Yang and Weir, 1998). It has also been widely used in previous studies (e.g., Zhang and Zhang, 2013; Zhang et al., 2014; Zhang, 2014).

#### English vocabulary breadth test

The Academic Vocabulary Test (AVT; Pecorari et al., 2019) was used to measure students’ English vocabulary breadth in this study. This vocabulary task required the participants to match the definition with the most appropriate word. The task consisted of 19 items, with each item providing 3 definitions on the left and 6 words on the right. The participants were rewarded 1 point for each correct match and the maximum possible score was 57. AVT had a Cronbach’s alpha coefficient of 0.91, thereby validating its reliability.

#### English vocabulary depth test

Word Associates Test (WAT) developed by Read (1993, 1995, 1998) was adopted to measure English vocabulary depth. This vocabulary subtask required the participants to identify the collocation, synonymous, part-whole, and whole-part relationships between the stimulus word and eight potential associates. The test was composed of 40 stimulus words, each followed by a list of eight words, four of which were semantically related to the stimulus word while the other four were not. Each correct answer would be awarded 1 point, and no penalty would be given for giving wrong answers. The maximum possible score of WAT was 160. The test had a Cronbach’s alpha coefficient of 0.93 (Read, 1995), indicating it was a reliable instrument. WAT and its modified versions have also been widely used in many previous studies (e.g., Nurweni and Read, 1999; Staehr, 2009; Zhang and Koda, 2012; Lee, 2020), thereby proving its validity and reliability.

### Pilot results of the English vocabulary test

A total of 12 freshmen and sophomore students, which form a subsample of this study, were recruited from a Southern Chinese university. The pilot study (see Table 2) aimed to ensure that the instruments for the vocabulary knowledge test have acceptable levels of reliability, and to check the correlation between the AVT and WAT scores. K-R 21 was adopted to examine the internal consistency of the vocabulary test in an equivalent way (Alderson et al., 1995; Qian, 1998). The r value for AVT was 0.85, whereas that for WAT was 0.89, indicating that both vocabulary tests had acceptable reliability. The Pearson product–moment correlation also showed a strong, positive correlation between the AVT and WAT scores (*r* = 0.90, *p <* 0.01). Thus, the results showed both the AVT and WAT were suitable for our research context.

**Table 2:** Pilot study (*N* = 12): Means, standard deviations, and score ranges.

Test	MPS	*M*	*SD*	Score range
AVT	57	21.33	9.05	10–38
WAT	160	100.83	17.61	76–125

MPS, maximum possible score.

### Procedures for data collection

The first author contacted some university English teachers about their willingness to include their students in this study. Ethical approval was obtained from each university before the current study was conducted. Those English teachers who were willing to include their students in the study were briefed on the data collection procedures. The first author also modelled the procedures for administering the questionnaires and tests. Given that the study participants were attending English classes every week, the data were collected during their intact English classes. All the students were informed that their participation was voluntary and that they could choose to withdraw from this study at any time. They were also informed that the data would only be used for research purposes and would be kept confidential. The students were required to finish all the tests and questionnaires in one sitting. Specifically, the students were required to spend 40 min answering the English reading test, 15 min answering the questionnaires, 15 min answering the AVT, and 30 min answering the WAT. The reading test answer sheets were marked according to the marking criteria established by the National College English Test Committee, whereas the vocabulary knowledge tests were marked according to the officially published reference answers. All questionnaire data and test scores were inputted into SPSS files for analysis.

### Data analysis

Before the analysis, the data were screened to check out cases that should be removed. Mean imputation by using expectation maximization (EM) was applied on missing values of no more than 10% of the data. SPSS 24.0 was used for the data analysis. Firstly, an internal consistency test and confirmatory factor analysis (CFA) were performed to check the reliability and validity of the reading strategy questionnaire and reading motivation questionnaire. The Cronbach’s coefficient of the internal consistency test, for which a value more than 0.60 suggests acceptable reliability, was used to evaluate the reliability of the questionnaires (Gan et al., 2019, 2021). The criteria for a good model fit are also listed as follows: *x*^2^/df < 3, CFI > 0.90, RMSEA<0.08, SRMR<0.08 (Hu and Bentler, 1999; Hooper et al., 2008). Descriptive statistics, including means, standard deviation, skewness, and kurtosis, were reported with regard to Chinese EFL learners’ English reading motivation, self-regulated reading strategy use, English vocabulary knowledge, and English reading comprehension. Secondly, to assess the contributions of English reading motivation, self-regulated reading strategies, English vocabulary knowledge to English reading comprehension, multiple regression analyses were run with English reading motivation factors, self-regulated reading strategy factors, English vocabulary depth and breadth as predictors and the English reading comprehension as the outcome variable. In order to reveal the reading proficiency differences in the aforementioned relationship pattern, the top 27%, middle 46%, and bottom 27% of these students’ English reading test scores were confirmed to be significantly different (Ebel and Frisbie, 1986; Bai and Guo, 2019) so that they were divided into high, average, and low achievers in the reading test. Three separate multiple regression analyses were then performed to examine how reading motivation, strategies, vocabulary knowledge contribute to reading comprehension across the three groups.

## Results

### Grouping reading proficiency levels

Based on the overall CET-4 reading test scores, all the participants were grouped into three clusters: The first 27 percentile of participants was labeled as the high achievers (*n* = 147); the last 27 percentile of participants as the low achievers (*n* = 147); and the rest students were grouped as the average achievers (*n* = 249). The maximum possible score for CET-4 reading test is 35. The average means of reading test results of the three groups were 6.89 (*SD* = 2.46) for the low achievers, 14.33 (*SD* = 2.66) for the average achievers, and 24.49 (*SD* = 3.60) for the high achievers. Then a repeated measures analysis of variance (ANOVA) and a post-hoc test were used to compare the differences across the three groups. It was found that high achievers, average achievers, and low achievers were significantly different from each other in the CET-4 reading test scores.

We also examined whether there were some differences between the three groups in terms of age, gender, year of English reading learning. No significant difference was revealed, indicating the three groups of participants were homogeneous in these personal factors.

### Confirmatory factor analyses

Confirmatory factor analyses **(**CFAs) were performed to examine if the two validated questionnaires were suitable in our research context. The CFA results for the reading motivation questionnaire show that the original five-factor model fits the data well (*x*^2^/df = 2.38 < 3, *p <* 0.001, CFI = 0.95, RMSEA = 0.05, SRMR = 0.05). The Cronbach’s alpha coefficients of the five reading motivational sub-scales are 0.86 (reading efficacy), 0.84 (reading enjoyment), 0.81 (recognition), 0.67 (involvement), and 0.75 (compliance), indicating that each sub-scale has good internal consistency. The results of CFA also indicate that reading strategy measurement model fits the data well (*x*^2^/df = 1.95 < 3, *p* < 0.001, CFI = 0.91, RMSEA = 0.04, SRMR = 0.05), and the Cronbach’s alpha coefficients for the seven factors are 0.73 (planning), 0.73 (evaluating), 0.85 (monitoring), 0.60 (initial reading), 0.55 (identifying important information), 0.75 (integrating), and.69 (inference making). Clearly, the Cronbach’s alpha coefficients of the reading strategy factors were mostly above.60 except for identifying important information strategies’ coefficient of 0.55. The overall reliability estimate of reading strategy questionnaire was 0.92.

As shown in Table 3, the composite reliability (CR) values for both English reading motivation factors and reading strategy factors were all above the threshold value of 0.60 (Hair et al., 2010) except one strategy factor (i.e., Identifying important information). Although the AVE values of the two reading strategy factors (Evaluation and Identifying important information) were below 0.30, the convergent validity of the construct is still adequate so long as CR values are acceptable (Fornell and Larcker, 1981; Malhotra and Dash, 2011).

**Table 3:** Convergent validity of confirmatory factor analysis for the two instruments (*N* = 543).

Measures	Items	CR	AVE
Motivation: Reading efficacy	7	0.85	0.45
Reading enjoyment	6	0.83	0.46
Recognition	3	0.81	0.59
Involvement	3	0.68	0.41
Compliance	3	0.76	0.51
Strategy: Planning	6	0.76	0.35
Evaluation	8	0.74	0.28
Monitoring	10	0.86	0.38
Initial reading	3	0.61	0.35
Identifying important information	4	0.52	0.23
Integrating	4	0.76	0.44
Inference making	3	0.69	0.43

CR, composite reliability; AVE, average variance extracted.

### Overview of the students’ English reading motivation, strategies, vocabulary knowledge, and reading comprehension

Table 4 presents descriptive statistics for reading motivation, strategies, vocabulary knowledge, and reading comprehension. The table shows that skewness and kurtosis values range between −2 and 2, indicating that the variables are normally distributed (Bachman and Kunnan, 2005). Among the five sub-scales of English reading motivation, recognition (*M* = 2.71, *SD* = 0.77) scored the highest, followed by compliance (*M* = 2.66, *SD* = 0.62), involvement (*M* = 2.53, *SD* = 0.64), and reading enjoyment (*M* = 2.27, *SD* = 0.67). Reading efficacy (*M* = 2.21, *SD* = 0.58) was reported to be the lowest among the five reading motivation factors. Among the seven sub-scales that measure self-regulated reading strategy use, the participants reported the highest use frequency of identifying important information (*M* = 3.57, *SD* = 0.80), followed by integrating (*M* = 3.42, *SD* = 0.84), monitoring (*M* = 3.33, *SD* = 0.76), inference making (*M* = 3.26, *SD* = 0.92), initial reading (*M* = 3.06, *SD* = 1.05), and planning (*M* = 3.05, *SD* = 0.86). The participants reported the lowest use frequency of evaluating (*M* = 2.77, *SD* = 0.79). The average English vocabulary breadth test score (i.e., 19.47) was below the mid-point of the scale (i.e., 28.50 points), and the average English vocabulary depth test score (i.e., 93.78) was above the mid-point of scale (i.e., 80 points), and the average English reading comprehension test score (i.e., 15.06) was close to the mid-point of the scale (i.e., 17.50).

**Table 4:** Descriptive statistics for English reading motivation, self-regulated reading strategy use, English vocabulary knowledge scores, and English reading comprehension test scores (*N* = 543).

	Subscale	MPS	Mean	SD	Skewness	Kurtosis	α
Motivation	REF	4	2.21	0.58	−0.002	−0.38	0.86
	REN	4	2.27	0.67	0.07	−0.36	0.84
	REC	4	2.71	0.77	−0.27	−0.36	0.81
	INV	4	2.53	0.64	−0.20	−0.04	0.67
	COM	4	2.66	0.62	−0.26	0.27	0.75
Strategy	PLA	5	3.05	0.86	−0.40	0.06	0.73
	EVA	5	2.77	0.79	−0.26	0.29	0.73
	MON	5	3.33	0.76	−0.40	0.53	0.85
	INI	5	3.06	1.05	−0.35	−0.07	0.60
	IDE	5	3.57	0.80	−0.42	−0.03	0.55
	INT	5	3.42	0.84	−0.47	0.36	0.75
	INF	5	3.26	0.92	−0.24	−0.21	0.69
Vocabulary tests	VBT	57	19.47	6.11	0.35	0.59	
	VDT	160	93.78	17.25	−0.92	1.28	
Reading test	RT	35	15.06	7.13	0.38	−0.48	

MPS, maximum possible score; REF, reading efficacy; REN, reading enjoyment; REC, recognition; INV, involvement; COM, compliance; PLA, planning; EVA, evaluating; MON, monitoring; INI, initial reading; IDE, identifying important information; INT, integrating; INF, inference making; VBT, English vocabulary breadth test; VDT, English vocabulary depth test; RT, English reading comprehension test.

### Contributions of English reading motivation, reading strategies, vocabulary knowledge to reading comprehension

Table 5 reports the correlations among reading motivation, reading strategies, vocabulary knowledge and reading comprehension. Results show that all motivation factors and strategy factors are significantly and positively correlated with one another (0.162 ≤ r ≤ 0.632, *p* < 0.001). Except for the use of initial reading and identifying important information strategies, all strategy factors are significantly correlated with vocabulary breadth test scores, and reading comprehension test scores (0.127 ≤ r ≤ 0.297, *p* < 0.05). In addition, except for the use of initial reading, identifying important information, and inference making strategies, all strategy factors are significantly associated with vocabulary depth test scores (0.090 ≤ r ≤ 0.147, *p* < 0.05). The motivation factors are also significantly and positively correlated with both vocabulary breadth and depth test scores, and reading comprehension test scores (0.092 ≤ r ≤ 0.391, *p* < 0.05). Finally, reading comprehension test scores are significantly and positively associated with vocabulary breadth test scores (*r* = 0.414, *p* < 0.001) and vocabulary depth test scores (*r* = 0.398, *p* < 0.001).

**Table 5:** Zero-order correlations among Chinese university EFL students’ English reading motivation, self-regulated reading strategy use, English vocabulary knowledge scores, and English reading comprehension test scores.

	1	2	3	4	5	6	7	8	9	10	11	12	13	14	15
1.PLA	_														
2.EVA	0.575***	_													
3.MON	0.603***	0.566***	_												
4.INI	0.371***	0.425***	0.466***	_											
5.IDE	0.359***	0.294***	0.405***	0.383***	_										
6.INT	0.431***	0.510***	0.606***	0.379***	0.368***	_									
7.INF	0.410***	0.524***	0.516***	0.382***	0.364***	0.602***	_								
8.REF	0.498***	0.438***	0.555***	0.328***	0.198***	0.477***	0.414***	_							
9.REN	0.371***	0.459***	0.402***	0.285***	0.176***	0.399***	0.349***	0.538***	_						
10.REC	0.299***	0.250***	0.319***	0.162***	0.166***	0.262***	0.254***	0.429***	0.305***	_					
11.INV	0.414***	0.419***	0.503***	0.275***	0.208***	0.434***	0.400***	0.490***	0.632***	0.375***	_				
12.COM	0.404***	0.333***	0.380***	0.231***	0.209***	0.308***	0.253***	0.328***	0.361***	0.320***	0.464***	_			
13.VBT	0.250***	0.164***	0.297***	0.075	0.050	0.203***	0.127**	0.345***	0.217***	0.157***	0.235***	0.202***	_		
14.VDT	0.114**	0.090*	0.147**	0.056	−0.028	0.094*	0.061	0.238***	0.168***	0.092*	0.168***	0.190***	0.446***	_	
15.RT	0.179***	0.180***	0.257***	0.028	0.031	0.177***	0.154***	0.391***	0.319***	0.203***	0.271***	0.233***	0.414***	0.398***	_

***p < 0.001; **p < 0.01; *p < 0.05 (two-tailed); PLA, planning; EVA, evaluating; MON, monitoring; INI, initial reading; IDE, identifying important information; INT, integrating; INF, inference making; REF, reading efficacy; REN, reading enjoyment; REC, recognition; INV, involvement; COM, compliance; VBT, English vocabulary breadth test; VDT, English vocabulary depth test; RT: English reading comprehension test.

To explore the contributions of reading motivation, reading strategies, vocabulary knowledge to reading comprehension, multiple regression analyses were carried out. The results of multiple regression analyses (Model 1) showed that reading efficacy (β = 0.211, *p* < 0.01), reading enjoyment (β = 0.139, *p* < 0.01), vocabulary breadth (β = 0.206, *p* < 0.01), vocabulary depth (β = 0.226, *p* < 0.01) significantly and positively predict students’ reading comprehension, initial reading (β = −0.127, *p* < 0.01), significantly and negatively predict students’ reading comprehension, whereas recognition (β = 0.023, *p* > 0.05), involvement (β = −0.011, *p* > 0.05), compliance (β = 0.062, *p* > 0.05), planning (β = −0.081, *p* > 0.05), evaluating (β = 0.016, *p* > 0.05), monitoring (β = 0.084, *p* > 0.05), identifying important information (β = −0.005, *p* > 0.05), integrating (β = −0.051, *p* > 0.05), and inference making (β = 0.023, *p* > 0.05) did not. The R^2^ was 0.319, indicating that 31.9% of the variance in students’ reading comprehension could be explained by these five predictors. Furthermore, by using only the five significant predictors as independent variables, regression results (Model 2) showed that reading efficacy (β = 0.222, *p* < 0.01), reading enjoyment (β = 0.148, *p* < 0.01), vocabulary breadth (β = 0.210, *p* < 0.01), vocabulary depth (β = 0.233, *p* < 0.01), initial reading (β = −0.115, *p* < 0.01) were still significant, with the regression model presenting a slightly decreased R^2^ (i.e., 0.310), indicating that reading efficacy, reading enjoyment, vocabulary breadth, vocabulary depth and initial reading had significant influence on students’ reading comprehension. The results also showed that the strongest variable affecting reading comprehension in this study was vocabulary depth. Details of the two regression models can be found in Table 6.

**Table 6:** Regression models reporting unstandardized (B), standardized betas (β), standard error (SE), *t* and *p* values for predictors of English reading comprehension (*N* = 543).

Predictor	English reading comprehension				
Model 1	***B***	***SE***	***β***	***t***	***p***
Motivation: Reading efficacy	2.581	0.635	0.211	4.064***	0.000
Reading enjoyment	1.478	0.540	0.139	2.738**	0.006
Recognition	0.216	0.383	0.023	0.563	0.574
Involvement	−0.121	0.580	−0.011	−0.208	0.835
Compliance	0.711	0.494	0.062	1.441	0.150
Strategy: Planning	−0.668	0.419	−0.081	−1.594	0.112
Evaluating	0.143	0.462	0.016	0.310	0.757
Monitoring	0.793	0.536	0.084	1.478	0.140
Initial reading	−0.864	0.294	−0.127	−2.945**	0.003
Identifying important information	−0.043	0.374	−0.005	−0.116	0.908
Integrating	−0.431	0.436	−0.051	−0.988	0.323
Inference making	0.180	0.378	0.023	0.476	0.635
Vocabulary breadth	0.241	0.050	0.206	4.850***	0.000
Vocabulary depth	0.093	0.017	0.226	5.531***	0.000
*R*^2^ = 0.319, *F*_14, 528_ = 17.671, *p* < 0.001					
**Model 2 (Only significant predictors in Model 1 included)**	***B***	***SE***	***β***	***t***	***p***
Reading efficacy	2.715	0.557	0.222	4.875***	0.000
Reading enjoyment	1.579	0.458	0.148	3.449**	0.001
Initial reading	−0.783	0.261	−0.115	−3.005**	0.003
Vocabulary breadth	0.245	0.049	0.210	5.034***	0.000
Vocabulary depth	0.096	0.017	0.233	5.778***	0.000
*R*^2^ = 0.310, *F*_5, 537_ = 48.317, *p* < 0.001					

***p < 0.001; **p < 0.01.

Three separate multiple regression analyses were also conducted for low, average, and high achievers. For the high achievers, the results (see Model 1, Table 7) showed monitoring strategies (β = 0.275, *p* < 0.05) and vocabulary depth (β = 0.286, *p* < 0.01) significantly and positively predicted students’ reading comprehension, whereas the rest of the independent variables did not. The R^2^ was 0.200, suggesting that 20.0% of the variance in high achievers’ reading comprehension could be explained by these two variables. In addition, by using only the two significant predictors as independent variables, regression results (see Model 2, Table 7) showed that monitoring strategies (β = 0.248, *p* < 0.01) and vocabulary depth (β = 0.264, *p* < 0.01) were still significant with a slightly decreased R^2^ (i.e., 0.140), suggesting both monitoring strategies and vocabulary depth could significantly predict high achievers’ reading comprehension. The results also showed that vocabulary depth was a better predictor of high achievers’ reading comprehension when compared than monitoring strategies. For the average achievers, the regression results (see Model 1, Table 8) showed that the model consisting of reading motivation, reading strategies, vocabulary knowledge did not predict reading comprehension (*p* > 0.05). Similarly, for the low achievers, the regression results (see Model 1, Table 9) also showed that the model did not predict reading comprehension (*p* > 0.05).

**Table 7:** Regression models reporting unstandardized (B), standardized betas (β), standard error (SE), *t* and *p* values for predictors of high achievers’ English reading comprehension (*N* = 147).

Predictor	English reading comprehension				
Model 1	***B***	***SE***	***β***	***t***	***p***
Motivation: Reading efficacy	0.497	0.809	0.073	0.615	0.540
Reading enjoyment	0.465	0.653	0.082	0.712	0.478
Recognition	0.465	0.429	0.099	1.085	0.280
Involvement	−0.587	0.673	−0.102	−0.873	0.384
Compliance	−0.694	0.492	−0.131	−1.410	0.161
Strategy: Planning	−0.295	0.448	−0.069	−0.659	0.511
Evaluating	0.027	0.567	0.005	0.047	0.963
Monitoring	1.375	0.662	0.275	2.077*	0.040
Initial reading	0.293	0.365	0.083	0.803	0.423
Identifying important information	−0.589	0.454	−0.131	−1.297	0.197
Integrating	−0.242	0.496	−0.055	−0.488	0.626
Inference making	0.223	0.410	0.057	0.545	0.587
Vocabulary breadth	0.001	0.063	0.002	0.018	0.986
Vocabulary depth	0.080	0.024	0.286	3.406**	0.001
*R*^2^ = 0.200, *F*_14, 132_ = 2.363, *p* < 0.01					
**Model 2 (Only significant predictors in Model 1 included)**	***B***	***SE***	***β***	***t***	***p***
Monitoring	1.237	0.387	0.248	3.195**	0.002
Vocabulary depth	0.074	0.022	0.264	3.413**	0.001
*R*^2^ = 0.140, *F*_2, 144_ = 11.702, *p* < 0.001					

**p < 0.01; *p < 0.05.

**Table 8:** Regression models reporting unstandardized (B), standardized betas (β), standard error (SE), *t* and *p* values for predictors of average achievers’ English reading comprehension (*N* = 249).

Predictor	English reading comprehension				
Model 1	***B***	***SE***	***β***	***t***	***p***
Motivation: Reading efficacy	0.580	0.422	0.119	1.373	0.171
Reading enjoyment	0.421	0.330	0.105	1.275	0.204
Recognition	0.332	0.284	0.087	1.168	0.244
Involvement	−0.116	0.377	−0.027	−0.307	0.759
Compliance	0.133	0.355	0.028	0.376	0.707
Strategy: Planning	−0.094	0.295	−0.031	−0.320	0.749
Evaluating	0.171	0.298	0.052	0.575	0.566
Monitoring	0.325	0.376	0.089	0.865	0.388
Initial reading	−0.152	0.197	−0.060	−0.769	0.443
Identifying important information	0.223	0.243	0.068	0.915	0.361
Integrating	−0.099	0.304	−0.030	−0.326	0.745
Inference making	−0.557	0.273	−0.183	−2.039*	0.043
Vocabulary breadth	0.034	0.032	0.075	1.038	0.300
Vocabulary depth	0.012	0.011	0.073	1.033	0.303
*R*^2^ = 0.087, *F*_14, 234_ = 1.598, *p* > 0.05					

*p < 0.05.

**Table 9:** Regression models reporting unstandardized (B), standardized betas (β), standard error (SE), *t* and *p* values for predictors of low achievers’ English reading comprehension (*N* = 147).

Predictor	English reading comprehension				
Model 1	***B***	***SE***	***β***	***t***	***p***
Motivation: Reading efficacy	−0.057	0.503	−0.013	−0.113	0.910
Reading enjoyment	−0.214	0.466	−0.052	−0.459	0.647
Recognition	0.291	0.263	0.101	1.110	0.269
Involvement	0.110	0.463	0.027	0.237	0.813
Compliance	0.775	0.386	0.188	2.008*	0.047
Strategy: Planning	−0.430	0.349	−0.146	−1.231	0.220
Evaluating	0.242	0.362	0.077	0.670	0.504
Monitoring	0.052	0.386	0.017	0.136	0.892
Initial reading	0.250	0.229	0.110	1.089	0.278
Identifying important information	−0.541	0.299	−0.174	−1.811	0.072
Integrating	0.481	0.335	0.170	1.437	0.153
Inference making	−0.427	0.276	−0.169	−1.547	0.124
Vocabulary breadth	0.018	0.043	0.039	0.411	0.682
Vocabulary depth	0.011	0.012	0.081	0.909	0.365
*R*^2^ = 0.117, *F*_14, 132_ = 1.255, *p* > 0.05					

*p < 0.05.

## Discussion

This study aimed to examine the contributions of English reading motivation, self-regulated reading strategies, and vocabulary knowledge to reading comprehension in the context of Chinese university EFL learners. The study confirmed five types of English reading motivation: reading efficacy, reading enjoyment, recognition, involvement, and compliance. The results indicated that Chinese university EFL students were generally extrinsically motivated to read English. This result is congruent with prior research reporting that L2 readers mainly read for extrinsic reasons (Dhanapala, 2008; Kim, 2011). One possible explanation is that university students tend to treat English reading as a way to reach their instrumental goals, such as pursuing further studies, seeking jobs, and improving their social status (Kim, 2011; Lin et al., 2012). Meanwhile, the students also reported a low level of reading efficacy. It might be that compared with their mother tongue, these students may encounter more obstacles when learning English reading because they commonly adopt Chinese as a communication medium in their learning activities (Lin et al., 2012).

This study also confirmed seven types of self-regulated reading strategies among Chinese university EFL students: planning, evaluating, monitoring, initial reading, identifying important information, integrating, and inference making. The results suggested that the students tended to use cognitive strategies more frequently in reading than they used metacognitive strategies, which echoes previous research that reported a preference of using cognitive strategies instead of using metacognitive strategies (e.g., Marton et al., 2005; Hung, 2014; Lau and Ho, 2016). Such preference may be associated with their learning environments in English reading classes (Lau and Ho, 2016). For example, teachers typically assume the role of an authority and offer few chances for student autonomy, preventing learners from becoming self-regulated readers in reading classes.

The regression analysis showed that reading enjoyment positively predicted students’ English reading comprehension. The result is aligned with the findings of previous studies suggesting the positive role of intrinsic motivation in L2 reading comprehension (e.g., Kim, 2011; Lin et al., 2012; Dhanapala and Hirakawa, 2016). In other words, students who were motivated by intrinsic motivation were likely to know the real sense of reading, seek valuable reading resources actively, adjust their reading pace, and feel encouraged and happy when facing reading challenges and solving difficulties (Reeve et al., 2012). It was also likely that these intrinsically motivated students might extend their reading to extracurricular reading activities, such as reading English newspapers, journals, novels, songs, and poems, which helped to contribute their reading comprehension.

Our results also showed that reading efficacy positively predicted students’ English reading comprehension, which is consistent with researchers’ earlier views (e.g., Shang, 2010; Irene, 2013; Boakye, 2015; Hwang, 2019) that students who believe that they can complete reading tasks successfully tend to achieve better reading results than those who possess negative beliefs about their reading competence. Owing to the importance of reading efficacy on reading comprehension, teachers need to assist students to build up their confidence in English reading by means of explicit strategy instruction, mastery experience development, and provision of positive feedback on students’ reading performance.

Unexpectedly, strategies of initial reading negatively predicted English reading comprehension, suggesting that students who used more of such strategies when taking reading tests would score lower in reading comprehension. Learning strategies, which involve a memory process, such as repeating, and confirming information, can negatively predict test performance (e.g., Purpura, 1997; Song, 2005). It could be possible that the students’ use of initial reading strategies in this study involved repeating and confirming information, which might result in negative influence on student reading comprehension. Zhang and Zhang (2013) also argued that if test takers spend more time focusing on the text itself instead of solving reading tasks, they may receive worse reading comprehension results.

Our research showed that both vocabulary depth and breadth positively predicted English reading comprehension, but vocabulary depth appeared to exert more powerful influence than vocabulary breadth. These results are in line with previous research findings which showed that vocabulary depth, rather than vocabulary breadth, plays a more important role in English reading comprehension (Kang et al., 2012). Our results thus provided further empirical evidence that vocabulary depth and breadth are two different and unique predictors of English reading comprehension (Qian, 1998). Consequently, in order to improve English reading comprehension, vocabulary knowledge should be developed from the perspectives of both breadth and depth.

Multiple regression analyses showed that the relationship pattern of the contribution of English reading motivation, self-regulated reading strategies, and vocabulary knowledge to English reading comprehension varied across high, average, and low achievers. For the low and average achievers, reading motivation, reading strategies, and vocabulary knowledge were found to have no significant positive influence on reading comprehension. However, for the high achievers, monitoring strategies and vocabulary depth significantly predicted reading comprehension in a positive way. The result that monitoring strategies predicted high achievers’ reading comprehension indicates that successful and less successful L2 readers differ in metacognitive strategy use (Rao et al., 2007; Zhang and Wu, 2009; Zhang, 2010). It might be that higher achievers’ metacognitive knowledge might assist them to make wise choices of when to read, what to read, how to read, why to read, and where reading strategies can be used to solve problems (Zhang, 2010). Consequently, use of metacognitive reading strategies such as monitoring will eventually lead to higher achievers’ improved reading comprehension. In contrast, lower-achieving readers may have insufficient metacognitive knowledge and find activating effective reading strategies difficult (Zhang, 2010). As a result, lower-achieving readers’ strategy use in the reading process may hardly enable them to effectively promote reading comprehension.

In this study, vocabulary depth was found to predict high achievers’ reading comprehension, but this was not the case for both low and average achievers. Again, it could be that higher-achieving readers’ better use of metacognitive strategies enabled them to grasp the meaning of reading materials better and faster, which eventually contributed to better reading outcomes (Carrell, 1989; Masrai, 2019).

## Conclusion and implications

This study examined how reading motivation, self-regulated reading strategies and English vocabulary knowledge influenced students’ English reading comprehension simultaneously in one model. The findings of this study offer pedagogical implications for assisting teachers in designing intervention aimed at improving their reading motivation, reading strategies, and vocabulary knowledge. Firstly, this study revealed that students generally maintained a relatively low level of intrinsic reading motivation. These students’ intrinsic reading motivation thus needs to be enhanced as our study has shown that reading enjoyment could positively predict English reading comprehension. To develop intrinsic reading motivation, teachers need to provide challenging and meaningful reading tasks, such as reading competitions, retelling stories, personal presentations based on reading texts, and role-playing games. Students may also actively participate in their favorite reading tasks and gradually develop their interests in English reading. Secondly, the students in this study reported a low level of English reading efficacy, indicating a pressing need to improve these students’ reading efficacy given that our study has also revealed a positive association between reading efficacy and reading comprehension. Teachers need to help students to build self-efficacy through mastery experience. For example, teachers can provide students with specific strategies they can use to facilitate their comprehension when they read (Tavakoli and Koosha, 2016). Providing positive feedback and praising students for improvement in English reading also facilitates development of reading efficacy. Thirdly, as monitoring strategies were found to positively predict high achievers’ reading comprehension, teachers should cultivate students’ use of self-regulated metacognitive strategies in the reading process by means of explicit metacognitive reading strategy instruction (Li et al., 2022). Finally, teachers should remind students of the importance of extending their vocabulary knowledge. Instead of adopting rote memorization in vocabulary learning, students should enhance their vocabulary in an authentic language environment.

This study extends our understanding of the role of English reading motivation, self-regulated reading strategies, and English vocabulary knowledge in students’ English reading comprehension. However, several limitations must be noted. First, the data on English reading motivation and strategies were self-reported by students in the questionnaires. Self-reported results may induce bias in social desirability (Bai and Wang, 2020). In future studies, some qualitative methods such as interviews, surveys, teachers’ classroom observations should be included for a more accurate reflection of students’ reading motivation and strategy use. Second, for the reading strategy questionnaire, some scales (e.g., initial reading, identifying important information) had lower estimates than some other scales. In future studies, those might be revised. Third, the sample was limited to first-year and second-year undergraduate students from five Chinese universities. Therefore, the findings of this work may not be generalized to other EFL contexts. Future studies should consider adopting large samples by including students from different types of universities, grade levels, and disciplines to test the generalizability of our findings. Fourth, given that the collected data were cross-sectional, this study was unable to generate cause-and-effect conclusions. A longitudinal or experimental design should be adopted in future studies to examine the causal associations.

## Data availability statement

The raw data supporting the conclusions of this article will be made available by the authors, without undue reservation.

## Ethics statement

The studies involving human participants were reviewed and approved by the University of Macau Ethics Assessment Committee. The patients/participants provided their written informed consent to participate in this study.

## Author contributions

HL and ZG conceived the idea and developed the material. HL implemented data collection and took the lead in writing the manuscript. ZG offered critical feedback and helped revise the manuscript. All authors have read and approved the manuscript.

## Conflict of interest

The authors declare that the research was conducted in the absence of any commercial or financial relationships that could be construed as a potential conflict of interest.

## Publisher’s note

All claims expressed in this article are solely those of the authors and do not necessarily represent those of their affiliated organizations, or those of the publisher, the editors and the reviewers. Any product that may be evaluated in this article, or claim that may be made by its manufacturer, is not guaranteed or endorsed by the publisher.
